# Transsacral colon fistula: late complication after resection, irradiation and free flap transfer of sacral chondrosarcoma

**DOI:** 10.1186/1477-7819-6-121

**Published:** 2008-11-11

**Authors:** Lars Steinstraesser, Michael Sand, Stefan Langer, Gert Muhr, Thomas A Schildhauer, Hans-Ulrich Steinau

**Affiliations:** 1 Department of Plastic Surgery, Burn Center, Hand Center, Sarcoma Reference Center Ruhr-University Bochum, Bergmannsheil, Bürkle-de-la-Camp Platz 1, 44791 Bochum, Germany; 2 Department of Surgery, Ruhr-University Bochum, Bergmannsheil, Bürkle-de-la-Camp Platz 1, 44791 Bochum, Germany

## Abstract

**Background:**

Primary sacral tumors are rare and experience related to accompanying effects of these tumors is therefore limited to observations on a small number of patients.

**Case presentation:**

In this case report we present a patient with a history of primary sacral chondrosarcoma, an infection of an implanted spinal stabilization device and discuss the challenges that resulted from a colonic fistula associated with large, life threatening abscesses as late complications of radiotherapy.

**Conclusion:**

In patients with sacral tumors enterocutaneous fistulas after free musculotaneous free flaps transfer are rare and can occur in the setting of surgical damage followed by radiotherapy or advanced disease. They are associated with prolonged morbidity and high mortality. Identification of high-risk patients and management of fistulas at an early stage may delay the need for subsequent therapy and decrease morbidity.

## Background

Primary sacral tumors are rare and experience related to accompanying effects of these tumors is therefore limited to observations on a small number of patients [1,2]. These include individuals with benign neoplasms such as osteochondroma, giant cell tumors and osteoid osteomas and, more commonly chordoma, myeloma, osteosarcoma and chondrosarcoma [3-6].

Sacral neoplasms cause mild but noticeable symptoms at an early stage. In these cases it is essential to achieve the right diagnosis in time for wide excision margins. A radical surgical approach with partial or total sacrectomy, including sacrifice of sacral roots and spinal-pelvic fixation, is technically challenging and may jeopardize axial stability. Surgical approaches are therefore often limited by the size of the tumor and additionally dictated by the proximity to vital structures. As a consequence a resection in sano is feasible only up to a certain size of the tumor.

By the time of diagnosis sacral tumors are often too large for achieving adequate margins. Although chondrosarcomas are reported to have low radio-sensitivity, local control is sometimes achieved through radiation in patients who have not been radically resected [7-9]. Nonetheless radiation-induced damage can cause major early and late post-radiation side effects, requiring management by the plastic surgeon [10,11]. Spinal stabilization devices, which are commonly used after resection of large sacral tumors, can become infected. After control of sepsis, wound drainage and debridement myocutaneous flaps enable long-term spinal stabilization and in some cases salvage of the implanted stabilization devices can be achieved [12].

In this case report we present a patient with a history of primary sacral chondrosarcoma, an infection of the implanted spinal stabilization device and discuss the challenges that resulted from a colonic fistula associated with large, life threatening abscesses as late complications of radiotherapy.

## Case presentation

**Figure 1:**
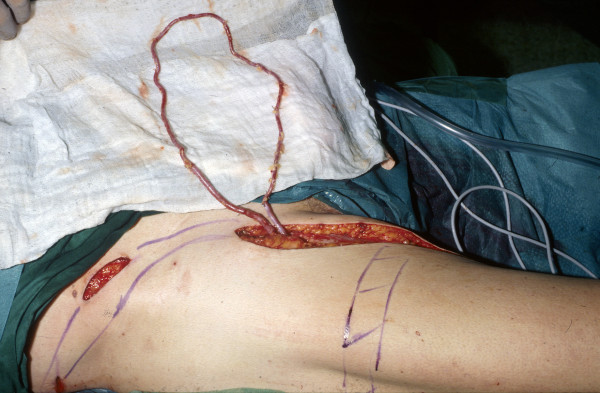
Saphenous vein graft anastomosed end-to-side to the superficial femoral artery (arterio-venous loop).

**Figure 2:**
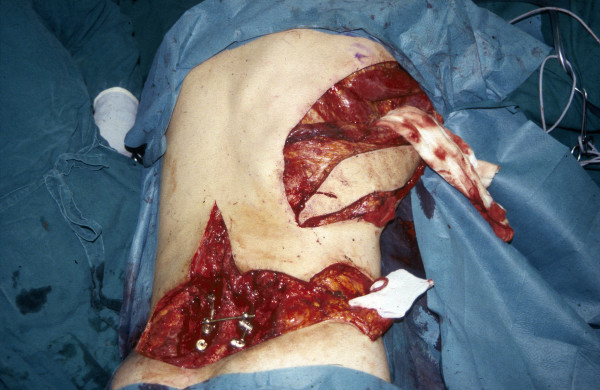
Latissimus dorsi free flap with the superficial femoral artery (arterio-venous loop) at the bottom right corner and an implanted Universal Spine System with fixation at the arcus root of L4, L5 and the lateral mass of the sacrum for stabilization at the bottom left corner.

A 57-year-old man with a history of chondrosarcoma of the Os sacrum was treated 1985 by a R1 resection at another institution. To support stabilization, implantation of an Universal Spine System (USS, Synthes, Inc., West Chester, PA) was followed by osteosynthesis of L4/pelvis and additional spongiosaplasty with fibula chips. Post-operatively neutron irradiation was started due to intralesional surgical margins. Ten years later (1995), the patient developed a fulminant osteomyelitis ending up with soft tissue defect of 38 × 26 cm. Following radical debridement with removal of both iliac crests, the fibula chips and the USS system. After extended wound treatment a new USS system with fixation at the arcus root of L4, L5 and the lateral mass of the sacrum was implanted for stabilization of the sacrum. The large lumbal soft tissue defect with exposed vertebrae and hardware was covered with a free flap. For vascular supply the complete saphenous vein graft (76 cm long) was served as an arterio-venous loop and was anastomosed end-to-side to the superficial femoral artery as previously described (Fig 1) [13]. The large wound was covered with a latissimus dorsi free-flap and anastomosed to the AV-loop (Fig 2). After uneventful postoperative period the patient could be discharged and the wound conditions have remained stable for over 10 years with a small fluid drainage from a cephalic sinus.

**Figure 3:**
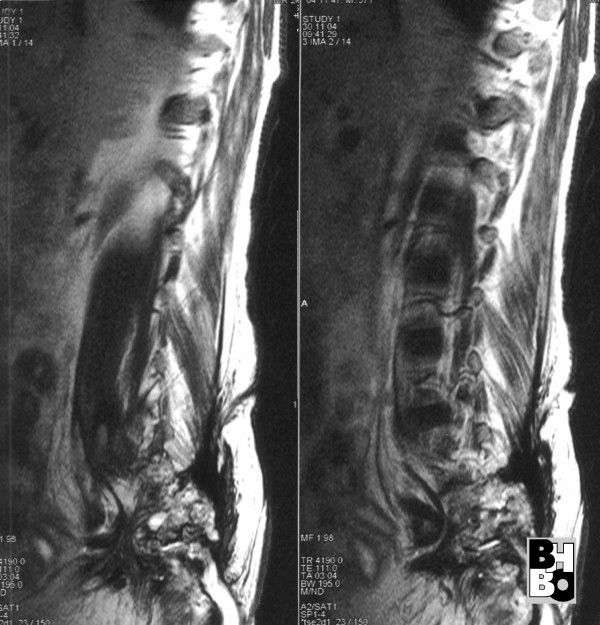
Sagital sections (MRI) of the lumbal and sacral spine showing extended osseous destructions of the sacrum.

**Figure 4:**
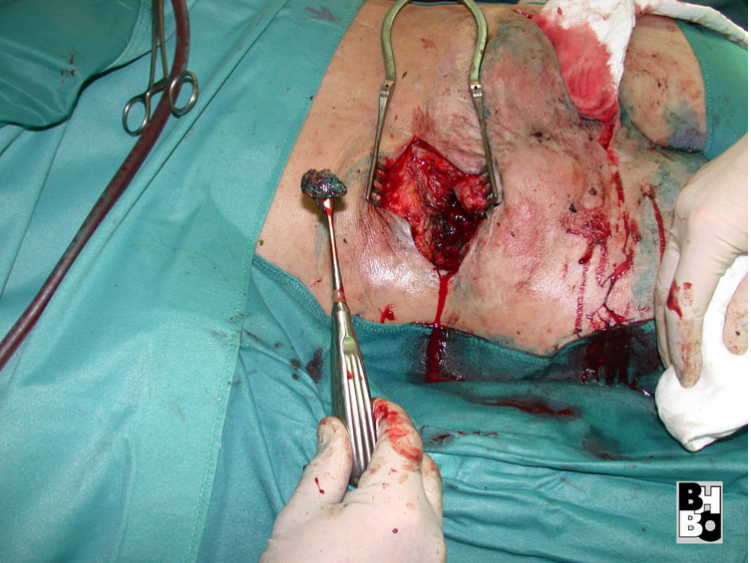
Sequestrectomy after methylene blue dye injection into the sacral fistulas.

In 2004 the patient presented with a recurrent inguinal hernia on the left side outside of our sarcoma center. The Shouldice repair of his hernia was uneventful. Three weeks after the operation hematological laboratory findings and blood chemistry values showed signs of infection. A CT-scan of the abdomen and pelvis showed a massive inflammatory infiltrate with air trapping contiguous to a large abscess in the right iliacal muscle (7 × 5 cm) and a collateral infiltrate of the psoas muscle. Additionally extended osseous destructions of the sacrum were documented. To differentiate between postoperative defects, the previously diagnosed sequestrating chronic osteomyelitis or a possible relapse of his chondrosarcoma was hardly possible (Fig 3). After antibiotic treatment of a urinary tract infection the patient developed an antiobiotic-associated diarrhea. The patient was then referred to us for further therapy of his life threatening hematogenous dissemination of bacteria from his multiple abscesses. By the time of referral in addition to the previously described ilacal and psoas abscesses he had developed an active discharging praesternal/mediastinal abscess and bilateral intracarpal infections. The abscesses were surgically drained and a bacterial smear revealed *Escherichia coli *in massive numbers. Antibiotic therapy was initiated with Imipenem and Metronidazol. Methylene blue dye was injected into the sacral fistulas, and multiple fistulectomys and a sequestrectomy were performed (Fig 4).

**Figure 5:**
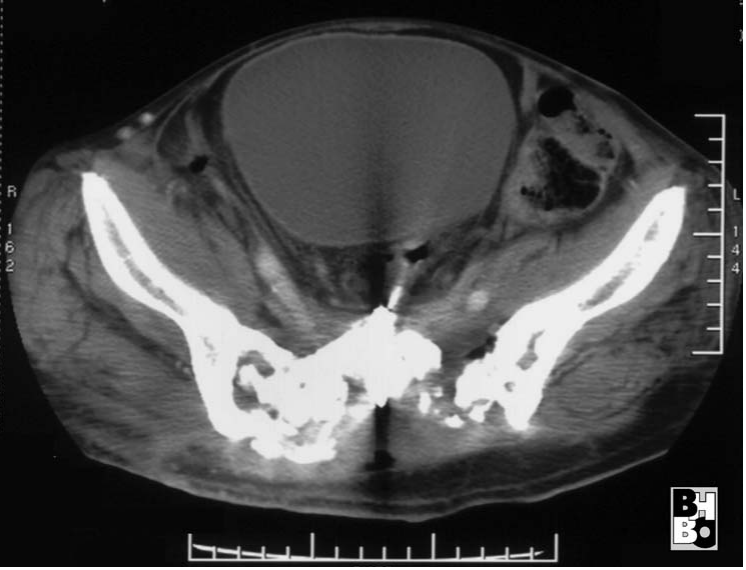
Gastrographin enema showing a gastrointestinal perforation reaching to the sacral lacunae.

**Figure 6:**
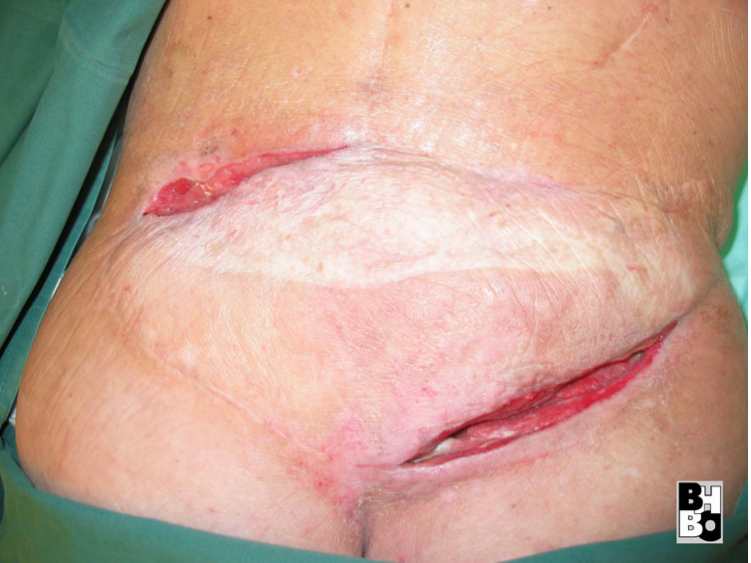
Latissimus dorsi free flap with fistula.

After ensuring sufficient dorsal drainage a gastrographin enema was performed to determine the site of a possible gastrointestinal perforation which was suspected as a possible source of infection. The contrast medium was shown to be leaking from the ascending colon and the caecum into the iliacal and psoas muscle, reaching to the sacral lacunae (Fig 5). A right hemicolectomy was performed. During the operation a perforation of the dorsal wall and the basis of the caecum were found. After clearing out a fecal abscess drainage was established and covered by split omentum-plasty followed by multiple rinses, using polymeric biguanide-hydrochloride (Lavasept^®^, Fresenius Kabi AG, Bad Homburg, Germany). Additionally the sacral and praesternal abscesses were once more debrided in the operating room. The right ureter was adherent to the abscess formation and was mobilized. Postoperatively the patient was referred to an intensive care unit. The bilateral septic inflammations of the carpal joints were successfully treated with high dose antibiotics after surgical excision and drainage (Imipenem and Metronidazol). After multiple irrigations of the large wounds and decreasing inflammation, granulation tissue developed. In order to further minimize the dead space of the large wounds, microdeformational wound therapy by means of Vacuum Assisted Closure (V.A.C.-Therapy^®^, KCI Medizinprodukte GmbH, Wiesbaden, Germany) was applied to the bilateral sacral wounds. Despite four weeks of intensive care his latissimus dorsi free flap was saved and the patient was discharged with wounds showing no signs of infection and a tendency towards good granulation (Fig 6). A control MRI in 2008 showed a stable fistula with no signs of recurrence.

### List of used products

Universal Spine System (USS, Synthes, Inc., West Chester, PA)

Polymeric biguanide-hydrochloride (Lavasept^®^, Fresenius Kabi AG, Bad Homburg, Germany)

Vacuum Assisted Closure (V.A.C.-Therapy^®^, KCI Medizinprodukte GmbH, Wiesbaden, Germany)

## Discussion

In patients with sacral tumors enterocutaneous fistulas after free musculotaneous free flaps transfer are rare. They occur in the setting of surgical damage followed by radiotherapy or advanced disease and are associated with prolonged morbidity and high mortality. Identification of high-risk patients and management of fistulas at an early stage may delay the need for subsequent therapy and decrease morbidity [14].

As in our case, ulceration of the gut and development of a fistula is based on changes in the collagen tissues and particularly in vascular tissue of the gut [15]. The bowel mucosal lining cells divide roughly every 22 days which is very fast compared to the other types of human tissue. Red and white cell precursors are the only cells which divide faster. Therefore radiation poisoning affects these two systems more than others which can ultimately, even several years after radiation therapy, result in enterocutaneous fistulas with all the possible side effects discussed in this case report.

## Conclusion

In summary, we have described a 57-yr-old sacral chondrosarcoma patient with a transsacral colon fistula complicated by E. *coli *bacteremia and multiple extra-intestinal manifestations.

## Consent

Written informed consent was obtained from the patient for publication of this case report and any accompanying images. A copy of the written consent is available for review by the Editor-in-Chief of this journal.

## Competing interests

All authors hereby disclose any commercial associations which might pose or create a conflict of interest with information presented in this manuscript. All authors declare that they have no competing interests.

## Authors' contributions

LS documented and prepared most of the draft. MS documented and prepared most of the draft. SL Literature research, revision of bibliography. GM Edited the manuscript and helped in preparing the draft. TAS Documented and prepared part of the draft. HUS Edited the manuscript, revision of bibliography and helped in preparing the draft. All authors read and approved final manuscript.

## References

[B1] Randall RL, Bruckner J, Lloyd C, Pohlman TH, Conrad EU (2005). Sacral resection and reconstruction for tumors and tumor-like conditions. Orthopedics.

[B2] Randall RL (2003). Giant cell tumor of the sacrum. Neurosurg Focus.

[B3] Deutsch H, Mummaneni PV, Haid RW, Rodts GE, Ondra SL (2003). Benign sacral tumors. Neurosurg Focus.

[B4] Biagini R, Orsini U, Demitri S, Bibiloni J, Ruggieri P, Mercuri M, Capanna R, Majorana B, Bertoni F, Bacchini P, Briccoli A (2001). Osteoid osteoma and osteoblastoma of the sacrum. Orthopedics.

[B5] Bergh P, Gunterberg B, Meis-Kindblom JM, Kindblom LG (2001). Prognostic factors and outcome of pelvic, sacral, and spinal chondrosarcomas: a center-based study of 69 cases. Cancer.

[B6] Leone A, Costantini A, Guglielmi G, Settecasi C, Priolo F (2000). Primary bone tumors and pseudotumors of the lumbosacral spine. Rays.

[B7] Rhomberg W, Eiter H, Böhler F, Dertinger S (2006). Combined radiotherapy and razoxane in the treatment of chondrosarcomas and chordomas. Anticancer Res.

[B8] Nedea EA, DeLaney TF (2006). Sarcoma and skin radiation oncology. Hematol Oncol Clin North Am.

[B9] Pritchard DJ, Lunke RJ, Taylor WF, Dahlin DC, Medley BE (1980). Chondrosarcoma: a clinicopathologic and statistical analysis. Cancer.

[B10] Novak JM, Collins JT, Donowitz M, Farman J, Sheahan DG, Spiro HM (1979). Effects of radiation on the human gastrointestinal tract. J Clin Gastroenterol.

[B11] Albu E, Gerst PH, Ene C, Carvajal S, Rao SK (1990). Jejunal-rectal fistula as a complication of postoperative radiotherapy. Am Surg.

[B12] Hultman CS, Jones GE, Losken A, Seify H, Schaefer TG, Zapiach LA, Carlson GW (2006). Salvage of infected spinal hardware with paraspinous muscle flaps: anatomic considerations with clinical correlation. Ann Plast Surg.

[B13] Germann G, Steinau HU (1996). The clinical reliability of vein grafts in free-flap transfer. J Reconstr Microsurg.

[B14] Chamberlain RS, Kaufman HL, Danforth DN (1998). Enterocutaneous fistula in cancer patients: etiology, management, outcome, and impact on further treatment. Am Surg.

[B15] Novak JM, Collins JT, Donowitz M, Farman J, Sheahan DG, Spiro HM (1979). Effects of radiation on the human gastrointestinal tract. J Clin Gastroenterol.

